# P-535. Implementation of Injectable Cabotegravir/Rilpivirine for Treatment of HIV in Patients with Substance Use Disorders at a Syringe Exchange Clinic

**DOI:** 10.1093/ofid/ofae631.734

**Published:** 2025-01-29

**Authors:** Amanda E Perez, Shane Nieves, Jessica Meisner

**Affiliations:** Hospital of the University of Pennsylvania, Phialdelphia, Pennsylvania; Prevention Point Philadelphia, Philadelphia, Pennsylvania; University of Pennsylvania Perelman School of Medicine, Philadelphia, Pennsylvania

## Abstract

**Background:**

Injectable Cabotegravir + Rilpivirine (CAB+RPV-LA) is a long-acting anti-retroviral therapy (ART) that has the potential to be utilized for people living with HIV (PLWH) who face significant barriers to daily ART. A key population that can benefit is PLWH with concomitant opioid use disorder (OUD) and housing instability. In this case series, we describe the implementation of a program that provides low-barrier access to CAB+RPV-LA for PLWH and OUD.

**Figure:**
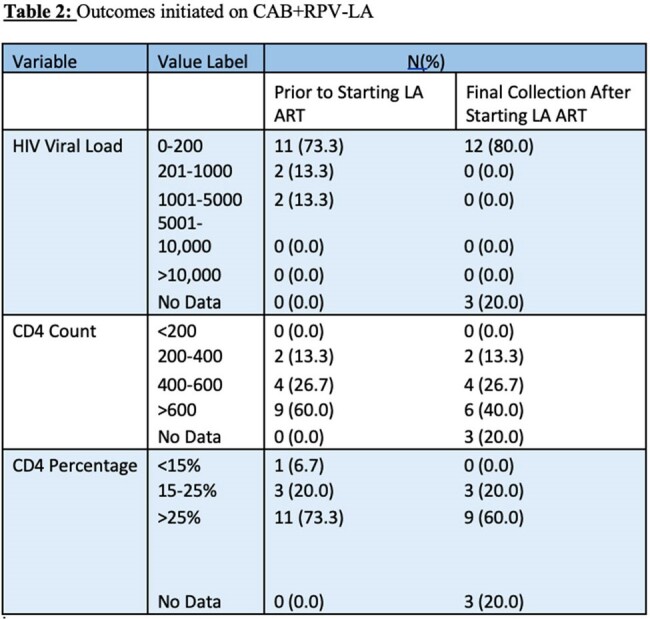

**Methods:**

The electronic medical record (EMR) was reviewed to identify individuals aged 18 years or older with a documented HIV diagnosis and a history of intravenous drug use (IVDU) who were initiated on CAB+RPV-LA between June 2022 - January 2024 at the Sana Clinic at Prevention Point Philadelphia (PPP). Fifteen patients were identified as having initiated CAB+RPV-LA during this time period. Descriptive statistics were used to summarize demographics, clinical characteristics, and outcomes of the study population.

**Results:**

A review of the patients started on CAB+RPV-LA found that 100% of patients (n =12) with follow-up labs after initiation had HIV viral load suppression and CD4 counts > 200. Three patients did not yet have follow-up labs since initiation due to their recent start on CAB+RPV-LA. Viral load on initiation ranged from undetectable to 1440. There were no missed doses during the two-year period. All patients had OUD and 67% had housing instability.

**Conclusion:**

Overall, CAB+RPV-LA was effective in maintaining and obtaining viral suppression in PLWH and OUD. To our knowledge, there is currently no literature on the implementation of long-acting ART for HIV maintenance in patients with OUD at a syringe exchange. Our findings support that long-acting ART is a key tool in ending the HIV epidemic especially at clinics serving patients with comorbid substance use and housing instability.

**Disclosures:**

**All Authors**: No reported disclosures

